# THE RELATIONSHIP BETWEEN OLDER DRIVERS’ RESILIENCE AND SELF-REPORTED DRIVING MEASURES OVER 5 YEARS

**DOI:** 10.1093/geroni/igz038.1245

**Published:** 2019-11-08

**Authors:** Renée M St Louis, Judith Charlton, Sjaan Koppel, Lisa J Molnar, Marilyn Di Stefano, Peteris Darzins, Michel Bédard, Shawn Marshall

**Affiliations:** 1 Monash University Accident Research Centre, Clayton, Victoria, Australia; 2 University of Michigan Transportation Research Institute, Ann Arbor, Michigan, United States; 3 VicRoads, Melbourne, Victoria, Australia; 4 Eastern Health Clinical School, Monash University, Forest Hill, Victoria, Australia; 5 Lakehead University, Thunder Bay, Ontario, Canada; 6 University of Ottawa/ Ottawa Hospital, Ottawa, Ontario, Canada

## Abstract

As people age into older adulthood, they are more likely to experience events that impact their driving, such as age-related cognitive and functional declines, serious illness, or disability. The ability to demonstrate resilience following such adversity may influence one’s decisions and feelings about driving. This study investigated whether resilience of older drivers changes over time, and if relationships between resilience, gender, and self-reported driving-related abilities, perceptions and practices remain stable or change. Participants were from the Candrive/Ozcandrive study, a prospective cohort study of older drivers from Canada, Australia and New Zealand. Analyses are presented from a subset of Ozcandrive participants (n=125) from Australia who completed a resilience scale at two time points approximately five years apart, as well as measures of driving comfort during the day and night, perceived driving abilities, and driving frequency. Participants were primarily male (67.2%) with a mean age of 81.6 years (SD=3.3, Range=76.0-90.0) at Time 1. Resilience increased significantly from Time 1 to Time 2 (Median=82.0/84.00, z=-2.9, p<.01). Although females had significantly higher resilience than males at both Time 1 (Median=84.0/81.0, U=2.3, p=.02) and Time 2 (Median=86.5/82.0, U=2.1, p=.03), there was a statistically significant increase in resilience of males over five years (p<.01) and no statistical change for females. Results show small but significant positive correlations, and increasingly stronger relationships over time between older drivers’ resilience and driving comfort as well as perceived driving abilities. Future research will use modelling to examine the association of various factors on the change in resilience and driving-related measures.

